# Risk of Surgical Site Infection in Posterior Spine Surgery Using Different Closing Techniques: A Retrospective Study of Two Neurosurgical Centers

**DOI:** 10.3390/jcm13247675

**Published:** 2024-12-16

**Authors:** Granit Molliqaj, Sara Lener, Michele Da Broi, Aria Nouri, Nalla Silva Baticam, Karl Schaller, Claudius Thomé, Pierre-Pascal Girod, Enrico Tessitore

**Affiliations:** 1 Division of Neurosurgery, Geneva University Hospitals, Rue Gabrielle-Perret-Gentil 4, 1205 Geneva, Switzerland; granit.molliqaj@hug.ch (G.M.); aria.nouri@hug.ch (A.N.); karl.schaller@hug.ch (K.S.); enrico.tessitore@hug.ch (E.T.); 2 Department of Neurosurgery, Innsbruck University Hospital, Anichstrasse 35, A-6020 Innsbruck, Austria; sara.lener@i-med.ac.at (S.L.); claudius.thome@i-med.ac.at (C.T.); dr.girod@neurospine.at (P.-P.G.); 3 Division of Neurosurgery, Quebec University Hospital, Laval University, 2705 Bd Laurier, Quebec, QC G1V 4G2, Canada; nalla.silva-baticam.med@ssss.gouv.qc.ca

**Keywords:** surgical complication, surgical site infection, spine, surgery

## Abstract

**Objectives:** To determine whether a closed dressing protocol reduces the surgical site infections (SSI) rate compared to conventional closing techniques. **Methods:** Patients who underwent lumbar spine surgery at two neurosurgical centers were retrospectively included from June 2015 to December 2019. Data on patients, general risk factors, and surgical risk factors for SSI were collected. Patients were subdivided into two groups: a Closed Protocol where the Dermabond^®^ ± Prineo^®^ dressing system was used, and a Conventional Protocol, namely sutures or staples. Statistical analysis was undertaken to compare the infection rates among the different closure techniques. **Results:** Altogether, 672 patients were included. In the whole cohort, 157 (23.36%) underwent skin closure with staples, 122 (18.15%) with sutures, 98 (14.58%) with intracutaneous sutures, 78 (11.61%) with Dermabond^®^, and 217 (32.29%) with Demabond^®^ + Prineo^®^. The overall infection rate was 2.23% (n = 15). Skin suture had the highest infection rate (4.10%), while the lowest was Dermabond^®^ (1.28%) and Dermabond^®^ + Prineo^®^ (1.4%), though the difference was not significant. Risk factors for SSI included higher BMI (29.46 kg/m^2^ vs. 26.96 kg/m^2^, *p* = 0.044), other sites infection (20.00% vs. 2.38%, *p* = 0.004), and a higher national nosocomial infections surveillance score (*p* = 0.003). **Conclusions:** This study showed that a closed protocol with the use of adhesive dressing with or without mesh had a slight tendency to lower infection rates compared to conventional protocol with sutures or staples, although no statistically significant difference was found between the closure techniques. Larger randomized studies are needed to investigate this potential benefit avoiding selection bias.

## 1. Introduction

Surgical site infection (SSI) is a frequent and dreaded complication in all surgeries. Not only does it generate discomfort for patients, but it also increases the costs associated with a possible risk of surgical revision, administration of antibiotics, and prolongation of the hospital stay. According to the literature, the rate of SSI in spinal surgery varies largely, ranging from 0.2% to more than 16%, and the incidence is higher in instrumented surgeries compared to those non-instrumented [1,2,3,4]. These remarkable differences depend on the type of operation and the patient’s risk factors, such as diabetes, smoking, immunosuppression, as well as many others [5]. For instance, preoperative antibiotic prophylaxis has clearly reduced the SSI rate [6]. Another factor that might play an important role in preventing infections is the skin closure technique. To date, different modalities of skin closure are known in spine surgery, such as skin sutures, staples, or, more recently, topical skin adhesives. Evidence in the literature about this topic is rather sparse [7]. Nevertheless, a water-tight skin closure seems to be an important element in reducing the risk of surgical site infection [4,8].

To the best of our knowledge, there are only a few studies in the literature comparing different closure techniques. A publication by Ando et al. in 2014 compared staples with 2-Octyl-Cyanoacrylate (Dermabond^®^; Ethicon Inc., Raritan, NJ, USA) “2-OCC” for wound closure. This study revealed that 2-OCC was associated with a lower rate of SSI than wound closure with staples. Moreover, the use of 2-OCC was more cost-effective in wound closure of 10 cm in length [9]. A more recent study was published by Johnston et al. in 2021, it included 11,911 patients and compared staple closure with waterproof wound dressings with 2-OCC plus polymer mesh tape (DERMABOND^®^; PRINEO^®^ Skin Closure System; Ethicon Inc., Raritan, NJ, USA; henceforth “2OPMT”) “2OPMT”. According to them, the 2OPMT was associated with a significantly lower operating room time, hospital length of stay, non-home discharge, and 90-day rates of infection or wound complications compared to skin staples [10]. A case series was published in 2014 and enrolled patients who underwent posterior spine surgery and for whom the 2-OCC was used for wound closure. They concluded that 2-OCC is a safe method for ultimate skin closure, without increased risk of wound-related complications. Anyhow, they did not compare the 2-OCC with other techniques [11].

According to the literature, Dermabond^®^ may reduce wound-related complications, such as SSI. With the present study, we wanted to compare skin sutures, staples, Dermabond^®^, and Dermabond^®^ plus Prineo^®^ as wound closure techniques with regard to SSI rate.

## 2. Methods

**Figure 1:**
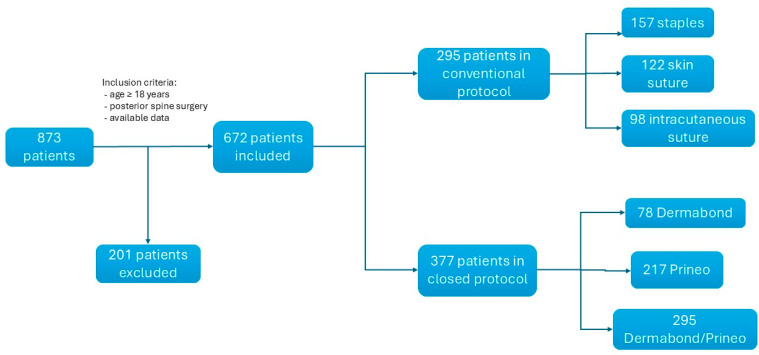
Flow diagram illustrating the patients’ selection process and the further subdivisions.

A retrospective study involving two neurosurgical centers (Geneva University Hospital, Switzerland, and Innsbruck University Hospital, Austria) was undertaken to assess the SSI rates of patients undergoing posterior spine surgery between June 2015 and December 2019. Research ethics board approval was received at both sites. All patients > 18 years old undergoing posterior spine surgery with complete data available were included (Figure 1). Data were retrospectively collected to assess the risk of infection, including age, sex, obesity, body mass index (BMI), smoking status, chronic corticosteroid treatment, and comorbidities, such as diabetes mellitus, cancer, immunosuppression, HIV status, and MRSA status. Data on the surgical procedure were also collected, including the type of surgical procedure (emergency, elective, or revision surgery), number of levels treated, duration of the procedure, blood loss, presence of dural tears, use of drain wound, type of wound closure, presence of wound infection (superficial or deep), and type of germ isolated. Finally, we collected data on general risk factors, such as the preoperative American Society of Anesthesiology (ASA) score, national nosocomial infections surveillance (NNSI) score, and surgical site infection risk score (SSIRS).

### 2.1. Institutional Antiseptic Procedure

As part of the institutional protocol, patients at both sites were instructed to shower using Betadine soap the morning prior to surgery. Hair removal was undertaken in the operating theatre if necessary. The antibiotic prophylaxis consisted of first-generation cephalosporin IV (Cefazoline 2 g) or Vancomycin 1 g in case of allergy. Furthermore, a supplementary half dose was administered every 4 h of surgery. Concerning skin disinfection, iodine solution or chlorhexidine was used on the surgical site for all surgical procedures. Finally, the skin was cleaned and disinfected again before closure.

### 2.2. Surgical Closure Techniques

According to the institutional standards, the fascia was closed using 1-CT suture (1-Circle Taper, Ethicon Inc., Raritan, NJ, USA) and subcutaneously with Vicryl 2-0 suture (Ethicon Inc., Raritan, NJ, USA). Regarding skin closure, multiple techniques were employed. Specifically, the closed protocol consisted of Dermabond^®^ plus Prineo^®^ (DBP) or Dermabond^®^ (DB) alone (Ethicon Inc., Raritan, NJ, USA), and the conventional protocol included staples (Appose^TM^ skin stapler, Medtronic, Minneapolis, MN, USA) intradermic suture (Vicryl rapide, Ethicon Inc., Raritan, NJ, USA), or 3-0 Prolene skin suture (Ethicon Inc., Raritan, NJ, USA). DB is a liquid adhesive containing a 2-OCC monomer, which polymerizes by drying and thus becomes more resistant and flexible [12]. Prineo^®^ is a polyester mesh that one places over the closure and facilitates wound edge approximation by providing an even distribution of tensile forces across the length of the incision. This device strengthens the suture and creates a sort of barrier that protects the wound. The combination of DB with Prineo^®^ is believed to result in better skin closure compared to DB alone. DB is applied with a pen applicator on the polyester mesh (Prineo^®^). Once the solution is dry, a patch of Opsite^®^ (Smith & Nephew, Watford, UK) might be applied to ensure sterility and reinforce watertightness.

### 2.3. Wound Monitoring

During hospitalization, the wounds were checked daily. After discharge, the follow-up was conducted at the local outpatient clinic according to the skin closure technique. Specifically, the removal of the DB or DBP was performed 14 days after the operation. With regard to staples or sutures, the wound dressing was removed every 2 days to observe the evolution of the wound, and the removal was undertaken between 10 and 14 days postoperatively. Superficial SSI was defined based on the NNIS program, which includes at least one of the following elements.

Based on the NNSI score defined, a superficial SSI is defined by at least one of these elements: (1) purulent discharge (culture documentation not required); (2) organisms isolated on fluid/tissue from the incision; (3) ≥1 signs of inflammation; (4) reopening of the wound by the surgeon; and (5) declaration of the wound as infected by the surgeon or a clinician. On the other hand, deep wound infection was identified if one of the following elements was present: (1) purulent drainage from the deep incision; (2) fascial dehiscence or fascia is deliberately separated by the surgeon because of signs of inflammation; (3) identification of a deep abscess at direct examination, during reoperation, by histopathology, or by radiologic examination; and (4) declaration of a deep wound infection by the surgeon or a clinician. For borderline cases of superficial SSI, where a deep wound infection could not be ruled out, we considered the case as a deep wound infection by default.

### 2.4. Statistics

Frequency distributions and summary statistics were calculated for all clinical and demographic data and independent t-tests were used to compare means. For categorical variables, cross-tabulations were generated, and Chi-square or Fisher exact tests were used to compare distributions. All statistical analyses were two-sided. A *p*-value < 0.05 was considered significant. IBM^®^ SPSS^®^ Statistics software version 27.0 (IBM^®^ Armonk, New York, NY, USA) was used for all statistical analyses.

## 3. Results

Altogether, this study included 672 patients who underwent a posterior surgical spine procedure with a total of 15 SSI resulting in an infection rate of 2.23% (Table 1).

### 3.1. Infection Cohort Characteristics

The infection cohort presented with a significantly higher BMI compared to those who had no postoperative infection (29.46 ± 6.96 kg/m^2^ vs. 26.96 ± 4.87 kg/m^2^, *p* = 0.044). Other site infections were also significantly more common in the infection group (20.00% vs. 2.38%, *p* = 0.004), as well as higher NNIS scores (*p* = 0.003).

A trend toward higher SSIRS (*p* = 0.058), longer surgeries (*p* = 0.079), and more consistent blood loss (*p* = 0.095) was highlighted in the infection cohort, even though this was not statistically significant.

Age at surgery, sex, and smoking status were similar to the whole cohort. According to our analyses, no statistically significant differences were found comparing the infection cohort with the whole cohort regarding surgical risk factors. Remarkably, the length of the surgical procedure, as well as dural tears, and the number of levels operated were not risk factors for SSI (Table 1).

### 3.2. Comparison Between the Conventional Protocol and Closed Protocol

Regarding patients’ characteristics, patients in the conventional protocol group were significantly older (62.47 ± 14.55 years vs. 59.76 ± 16.01 years, *p* < 0.05). BMI was also significantly higher compared to the closed protocol cohort (27.38 ± 5.26 kg/m^2^ versus 26.42 ± 4.26 kg/m^2^, *p* < 0.05), while the other variables were similar between the two groups.

With regard to comorbidities, in the conventional protocol group, more patients presented with cancer (5.31% vs. 1.35%, *p* < 0.01). Otherwise, no statistically significant difference between the two groups was detected.

Concerning risk factors for infection, conventional protocol patients had poorer ASA score (*p* < 0.001). Specifically, only 7.69% (n = 29) had an ASA score of 1 compared to 15.25% (n = 45) in the closed protocol group, and more than 25% had an ASA score of 3 in the conventional protocol group in comparison to 17.29% (n = 51) in the other cohort (*p* < 0.001). SSIRS was also significantly higher in the conventional protocol group (2.49% vs. 1.43%, *p* < 0.001).

Regarding surgical factors, the conventional protocol was elected as a wound closure technique more frequently in emergency cases (11.94% vs. 6.78%, *p* < 0.05). The duration of surgery was significantly longer in conventional protocol patients (147.75 ± 82.86 min vs. 132.30 ± 90.05 min, *p* < 0.05). Furthermore, in the conventional protocol group, surgical wound drains were used significantly more frequently (47.21% vs. 36.61%, *p* < 0.01), and patients underwent more revision surgeries compared to the closed protocol cohort (6.37% vs. 0.68%, *p* < 0.001). Moreover, there was a tendency toward a higher frequency of dural tears in patients receiving conventional protocol, even though it was not statistically significant (8.75% vs. 5.08%, *p* = 0.067) (Table 1).

### 3.3. Infection Rate According to the Wound Closure Technique

Of the 672 patients included in this study, 157 (23.36%) received staples, 122 (18.15%) had skin sutures, 98 (14.58%) had intracutaneous closure, 78 (11.61%) received DB, and 217 (32.29%) had DBP (Table 2).

The highest infection rate was observed with skin sutures (n = 5, 4.10%), while the lowest was detected in the DB (1.28%) and DBP (1.36%) groups, respectively. Regarding the other techniques, in the staple group, the infection rate was 2.55% (n = 4) and 1.38% with Prineo only. Nonetheless, comparing each infection rate with the rest of the cohort, no statistically significant difference was observed (Table 2).

## 4. Discussion

To the best of our knowledge, this is the first study providing a direct comparison between common wound closure techniques, such as staples, skin sutures, and intracutaneous sutures, and novel techniques of wound adhesion, such as DB and DBP.

Wound healing-related complications are one of the most serious problems in lumbar spine surgery. Indeed, the consequences may be so devastating on the tissues that for complex cases, collaboration with plastic surgeons is of paramount importance. For instance, for cases where tissue defect is so conspicuous that reconstruction with a flap is needed. According to Adogwa et al., SSI was the most common primary reason for unplanned readmission in a cohort of 1400 patients [13]. Waterproof wound dressing might reduce SSI. However, at present, almost only low-quality evidence is available, and systematic reviews are frequently inconclusive. In 2020, Tan et al. [14] published a systematic review to determine the rate of SSI, wound dehiscence, and wound erythema with cyanoacrylate glue closure in spine surgery. Only five articles were included and four of them were case series. A total of 1282 patients were analyzed. They concluded that cyanoacrylate dermal closure for ACDF, posterior cervical decompression, lumbar microdiscectomy, and lumbar laminectomy are associated with lower rates of complications. Namely, they found 0.41% SSI in lumbar microdiscectomy and 1.82% in lumbar laminectomy. However, no comparative statistics were undertaken, and no data were available regarding instrumented spine surgery which, according to the literature, is the most affected surgical procedure [1]. Finally, Mostofi et al. in 2023 published an interesting article including a total of 4383 patients and compared different conventional closure techniques in lumbar spine surgery with regard to wound dehiscence, delayed healing, and wound infection. In the first place, the wound infection rate was rather similar for all closure techniques. Secondly, they did not consider new wound adhesion techniques in their study [15].

In our study, an overall infection rate of 2.23% (n = 15) was observed, which is in line with the rate of infection reported in the literature [1,16,17]. According to a recently published systematic review including 22,475 patients, the incidence of lumbar spine postoperative infection was 2.7%. Notably, a higher incidence was found in instrumented surgery compared to non-instrumented one (4.4% vs. 1.4%), and posterior approaches presented with more infections compared to anterior ones (5.0% vs. 2.3%) [1]. Lower infection rates are reported by Mostofi et al., namely 0.96% for the whole cohort. However, these results are not representative of the general population, since the authors declared that they intentionally excluded patients carrying other wound healing risk factors, such as diabetes, BMI lower than 18.5 kg/m^2^ or higher than 24.9 kg/m^2^, and intraoperative dural tears [15].

As described by other authors [17,18], we found that patients with infections showed significantly higher BMI compared to the whole cohort (26.96 ± 4.87 vs. 29.46 ± 6.96, *p* = 0.044), had other site infections (2.4% vs. 20.0%, *p* = 0.004), and presented with higher risk as assessed by NNIS score (*p* = 0.003). Notably, older patients, those with higher BMI or higher SSIRS scores, and oncologic patients were more commonly treated by conventional techniques for closure. In light of these findings, the presence of a selection bias must be considered. Specifically, surgeons may have the tendency to select ideal cases when they use the closed protocol, namely those with a lower BMI, fewer comorbidities, first surgery, elective cases, etc. This limitation must be taken into consideration when one looks at the infection rates by closure techniques since the patients do not have the same characteristics.

Surprisingly, no difference in terms of surgical factors was found comparing the infection cohort with the whole cohort. However, this lack of significance is more likely explained by the small size of the infection cohort, namely only 15 patients. On the other hand, patients who received the conventional protocol were significantly more often operated in an emergency setting (6.78% vs. 11.94%), had longer operations (132.30 ± 90.05 vs. 147.75 ± 82.86, *p* < 0.05), had a significantly worse preoperative ASA score (*p* < 0.001), and higher SSIRS scores (*p* < 0.001). These differences are also potentially related to a selection bias and may influence the results regarding infection rates by wound closure technique. In fact, these are well-documented risk factors for wound infection in lumbar spine surgery [4,15].

Interestingly, most revision surgeries were closed using the conventional protocol (0.68% vs. 6.37%, *p* < 0.001). Indeed, the best results in terms of wound closure with the closed protocol are achieved when the wound margins are regular and perfectly accommodated, which is rarely the case in a revision setting. Hence, it is often the surgeon’s preference to close using a conventional technique after a revision, given that staples or sutures allow a better correction of the irregularities of the wound.

Of note, significantly more patients had a wound drain after surgery in the conventional protocol cohort compared to the closed protocol (36.61% vs. 47.21%, *p* < 0.01). According to the literature, there is still no evidence substantiating a higher SSI rate associated with drain placement [8,19] and this practice is strongly dependent on the center’s habits. The difference between the two cohorts might be partially due to the selection bias mentioned above. Indeed, revision surgery, higher BMI, and oncologic patients may more frequently need a wound drain after surgery because intraoperative bleeding is harder to control or the wound closure is more complex. Furthermore, the balance between any potential increased risk of infection related to drains and their potential benefit of preventing fluid collection and potential neural compression remains unclear.

Some authors suggest that dural tears could also be a predicting factor for the development of SSI [20]. However, according to our analyses, no significant difference was found with respect to dural tears between the infection cohort and the whole cohort (7.14% vs. 6.67%, *p* = 1.000).

One of the potential advantages of the 2-OCC is that it may shorten closure time. According to our analysis, patients treated with DB or DBP had a statistically significant shorter length of surgery compared to the conventional protocol. Shortening closure time may be impactful on the risk of infection since the wound stays open for a shorter period. Moreover, 2-OCC requires less postoperative care during the follow-up. This also might be associated with a lower risk of infection, as well as a reduction in health care costs compared to other skin closure techniques that need regular maintenance of the wound during the immediate postoperative period until removal of sutures or staples by a nurse or a physician (depending on the country). Another difference with the conventional protocol is that the application of DB/DBP does not require damaging the skin as one does with staples or sutures. Indeed, in order to put stitches or staples, the surgeon makes micro-damage on the skin and potentially brings into the wound bacteria, which live on the skin. Another hypothesis could be that in the closed-protocol group, proper closure of the subcutaneous tissue is required, as there is no opportunity to adjust the wound edges using adhesive. This step may be performed less cautiously when the surgeons know they can adapt the wound with skin sutures or staples. This might create small loose areas in the wound, which allow germs to enter the soft tissues during the early postoperative days. Hence, we deem that 2-OCC could be a valid option when spine surgeons deal with long incisions without damaged wound margins. Indeed, this technique could reduce surgical time, as well as the risk of infection. Unfortunately, these theories are not confirmed by the present article and further studies are needed. Indeed, even though we showed that DB and DBP had the lowest infection rates, this finding was not statistically significant. Given that other recent studies have shown a significant reduction in infection rate with DB, our lack of significance might be due to the limited number of infected cases in our cohort [9,10,11,21].

Furthermore, closure with a closed protocol seems to be more comfortable for patients. Indeed, DB and DBP require less manipulation compared to the standard closure techniques. Moreover, these systems are waterproof, and patients are allowed to have a shower soon after the procedure. Of course, these are only speculations, and further studies involving patients’ surveys and evaluation of patients’ scars or postoperative pain due to the dressing are needed to objectify these considerations.

## 5. Strengths and Limitations

This study has several limitations. In the first place, given the limited number of infected cases, a larger cohort of SSI or the comparison with a matched cohort with similar characteristics would have been ideal to show statistically significant differences. Therefore, despite the relatively important size of the cohort, the present study remains probably underpowered. Secondly, the conventional protocol group had more common important risk factors for postoperative infection, such as older age, higher BMI, cancer, poorer ASA score, and other surgical risk factors. As repeatedly highlighted in the manuscript, these discrepancies are likely due to a selection bias introduced intraoperatively by surgeons who decided to use a conventional closure technique instead of a new one based on the case. In order to overcome this potential bias, a randomized controlled design should be elected. Finally, multivariate analysis in order to rule out the confounding factors effect could not be performed due to the restricted amount of infections in our cohort.

Regarding the strengths of our study, this represents one of the few publications addressing the question of whether there is a difference in terms of SSI by closure techniques, whereas other studies do not take into account all closure techniques. Indeed, we compared four different closure types in a relatively large cohort of more than 600 patients. Moreover, this is a multicenter study involving two centers and several surgeons, which offers a more accurate representation of infection rates by closure techniques.

## 6. Conclusions

This study showed that a closed protocol with the use of adhesive dressing with or without mesh had a slight tendency to lower infection rates compared to the conventional protocol with sutures or staples, although no statistically significant difference was found between the closure techniques. Larger randomized studies are needed to investigate this potential benefit avoiding selection bias.

## Figures and Tables

**Table 1:** Patient demographics, comorbidities, risk factors, and surgical factors by surgical site infection and closure protocol.

Patient Demographic	Total Cohort (n = 672)	Infection Cohort (n = 15)	*p*-Value	Closed Protocol (n = 295)	Conventional Protocol (n = 377)	*p*-Value
Age (years)	61.32 ± 15.33	63.2 ± 11.3	NS	59.76 ± 16.01	62.47 ± 14.55	<0.05
Sex (F, %)	44.64% (n = 300)	46.67% (n = 7)	NS	45.42% (n = 134)	44.03% (n = 166)	NS
BMI (kg/m^2^)	26.96 ± 4.87	29.46 ± 6.96	<0.05	26.42 ± 4.26	27.38 ± 5.26	0.01
Active Smoking (Y, %)	28.57% (n = 192)	26.67% (n = 4)	NS	28.14% (n = 83)	28.91% (n = 109)	NS
Comorbidities (n, %)						
HIV	0.45% (n = 3)	6.67% (n = 1)	NS	1.00% (n = 3)	0.00% (n = 0)	NS
Cancer	3.57% (n = 24)	6.67% (n = 1)	NS	1.35% (n = 4)	5.31% (n = 20)	<0.01
Diabetes	15.03% (n = 101)	6.67% (n = 1)	NS	15.25% (n = 45)	14.85% (n = 56)	NS
Immunsuppresion	2.68% (n = 18)	13.33% (n = 2)	NS	2.37% (n = 7)	2.92% (n = 11)	NS
Steroid treatment	2.53% (n = 17)	13.33% (n = 2)	NS	3.05% (n = 9)	2.12% (n = 8)	NS
MRSA status	0.30% (n = 2)	0.00% (n = 0)	NS	0.00% (n = 0)	0.68% (n = 2)	NS
Other site infection	2.38% (n = 16)	20.00% (n = 3)	<0.005	1.70% (n = 5)	2.92% (n = 11)	NS
Risk Factors						
ASA Score						
1	11.01% (n = 74)	0.00% (n = 0)	NS	15.25% (n = 45)	7.69% (n = 29)	<0.001
2	65.63% (n = 441)	60.00% (n = 9)		66.78% (n = 197)	64.72% (n = 244)	
3	22.92% (n = 154)	40.00% (n = 6)		17.29% (n = 51)	27.32% (n = 103)	
4	0.45% (n = 3)	0.00% (n = 0)		0.68% (n = 2)	0.26% (n = 1)	
NNIS						
0	68.16% (n = 458)	46.67% (n = 7)	<0.005	69.49% (n = 205)	67.11% (n = 253)	NS
1	28.42% (n = 191)	33.33% (n = 2)		26.44% (n = 78)	29.97% (n = 113)	
2	3.27% (n = 22)	20.00% (n = 3)		4.07% (n = 12)	2.65% (n = 10)	
3	0.15% (n = 1)	0.00% (n = 0)		0.00% (n = 0)	0.26% (n = 1)	
SSIRS	2.02% ± 2.85%	3.41% ± 3.20%	NS	1.43% ± 0.87	2.49% ± 3.67%	<0.001
Surgical Factors						
Emergency Surgery (Y)	9.67% (n = 65)	20.00% (n = 3)	NS	6.78% (n = 20)	11.94% (n = 45)	<0.05
Length of Surgery (min)	141.00 ± 86.36	197.87 ± 118.33	NS	132.30 ± 90.05	147.75 ± 82.86	<0.05
Dural Tear (Y)	7.14% (n = 48)	6.67% (n = 1)	NS	5.08% (n = 15)	8.75% (n = 33)	NS
Blood Loss (mL)	138.93 ± 241.12	313.33 ± 384.27	NS	121.90 ± 257.63	152.29 ± 226.81	NS
Placement of drain (Y)	42.56% (n = 286)	66.67% (n = 10)	NS	36.61% (n = 108)	47.21% (n = 178)	<0.01
Number of Surgical Levels	1.37 ± 1.07	2.40 ± 2.67	NS	1.31 ± 1.08	1.42 ± 1.06	NS
Revision Surgery (Y)	2.38% (n = 16)	0.00% (n = 0)	NS	0.68% (n = 2)	6.37% (n = 24)	<0.001

**Table 2:** Surgical site infection rate by closure technique.

	Staple Closure		Skin Suture Closure		Intracutaneous Closure		Dermabond		Prineo		Dermabond/Prineo	
	Conventional Protocol						Closed Protocol					
Closure technique	Staples 23.36% (n = 157)	Other 76.64% (n = 515)	Skin suture 18.15% (n = 122)	Other 81.85% (n = 550)	Intracutaneous suture 14.58% (n = 98)	Other 85.42% (n = 574)	DB 11.61% (n = 78)	Other 88.39% (n = 594)	Prineo alone 32.29% (n = 217)	Other 67.71% (n = 455)	DBP 43.90% (n = 295)	Other 56.10% (n = 377)
SSI rate	2.55% (n = 4/257)	2.14% (n = 11/515)	4.10% (n = 5/122)	1.82% (n = 10/550)	3.06% (n = 3/98)	2.09% (n = 12/574)	1.28% (n = 1/78)	2.36% (n = 14/594)	1.38% (n = 3/217)	2.64% (n = 12/455)	1.36% (n = 4/295)	2.92% (n = 11/377)
*p*-value	NS		NS		NS		NS		NS		NS	

## Data Availability

The data presented in this study are available on request from the corresponding author. The data are not publicly available due to ethical restrictions.

## Abbreviations

BMI	Body Mass Index
DBP	Dermabond^®^ plus Prineo^®^
DB	Dermabond^®^
NNSI	National Nosocomial Infections Surveillance
SSI	Surgical Site Infection
NS	Not Significant
